# Crystal structure of the nucleoside 2′-de­oxy­guanosine dimethyl sulfoxide disolvate

**DOI:** 10.1107/S2056989023007405

**Published:** 2023-08-30

**Authors:** Bernhard Spingler

**Affiliations:** a Department of Chemistry, University of Zurich, Winterthurerstrasse 190, Zurich 8057, Switzerland; University of Durham, United Kingdom

**Keywords:** crystal structure, nucleoside, guanine, guanosine, purine

## Abstract

The title com­pound, C_10_H_13_N_5_O_4_·2C_2_H_6_OS, which is of inter­est with respect to its biological activity, at 183 K has ortho­rhom­bic (*P*2_1_2_1_2_1_) crystal symmetry. The structure displays a network of inter­molecular N—H⋯N, N—H⋯O and O—H⋯O hydrogen bonds. 2′-De­oxy­guanosine mol­ecules are linked to each other and to the two dimethyl sulfoxide solvent mol­ecules by hydrogen bonding.

## Abstract 2

The first high-quality crystal structure of unmodified 2′-de­oxy­guanosine is reported. The isolated crystals are the dimethyl sulfoxide disolvate.

## 1. Chemical context

De­oxy­nucleosides are the building blocks of DNA, the storage place for the genetic information in most organisms. Understanding the properties of DNA is crucial for our knowledge of its reactivity in cellular processes of replication and transcription to yield transfer RNA (Stryer, 1995 ▸). Furthermore, mutagenic reagents can irreversibly alter the structure and function of DNA (Wang *et al.*, 1998 ▸). In view of all this, it is of upmost importance to know the precise geometric parameters of all the nucleobases. These parameters are needed for techniques such as macromolecular X-ray crystallography in some cases and (NMR restrained) modelling of oligonucleotides (Clowney *et al.*, 1996 ▸; Gelbin *et al.*, 1996 ▸). Surprisingly, no high-quality crystal structure of unmodified 2′-de­oxy­guan­osine has been published to date. In the course of studying the inter­action of nucleobases with copper(II) (Santangelo *et al.*, 2007 ▸), we obtained single crystals of 2′-de­oxy­guanosine as a solvate with two mol­ecules of dimethyl sulfoxide (DMSO), (**I**), and characterized it by X-ray diffraction. 
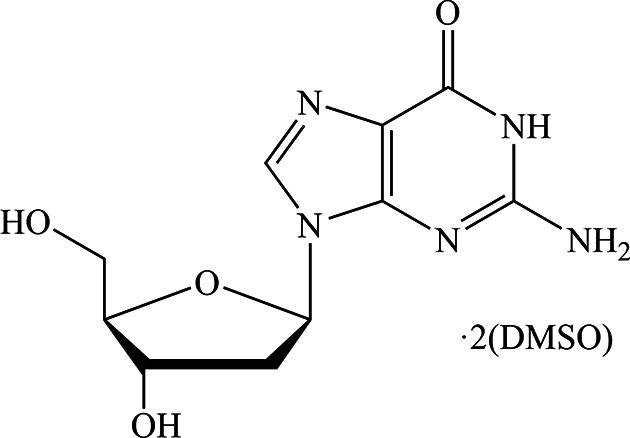


## 2. Structural commentary

**Figure 1:**
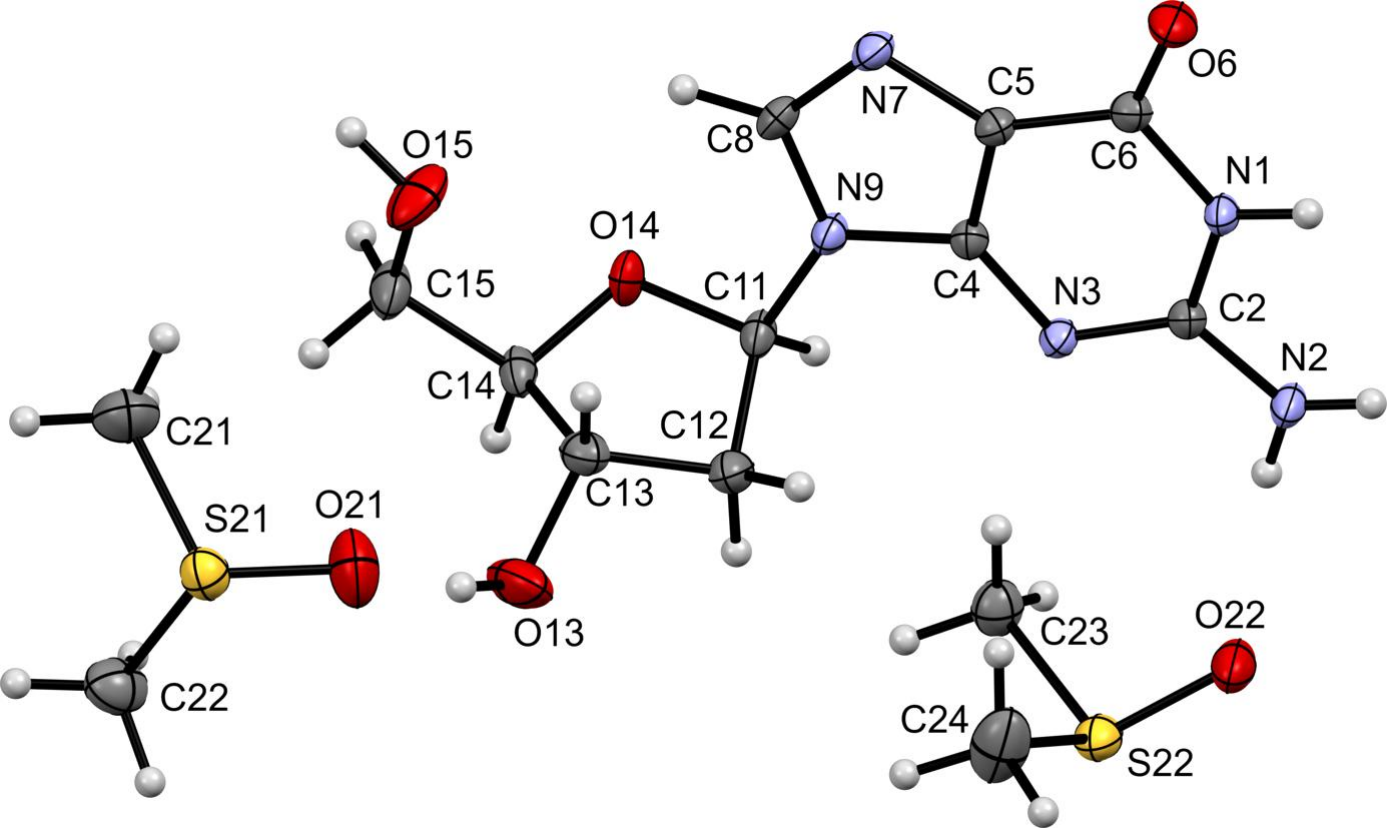
Displacement ellipsoid plot (50% probability) of (**I**).

Nucleobase (**I**) crystallized in the ortho­rhom­bic Sohnke space group *P*2_1_2_1_2_1_, with four formula units per unit cell and one per asymmetric unit (Fig. 1 ▸). The sugar conformation at the C3′ position (C13) is *endo*. The torsion angle χ (Alvarez *et al.*, 2019 ▸; Schabert *et al.*, 2021 ▸) of O14—C11—N9—C4 is −165.6 (1)° (Table 1 ▸). The freely refined H atoms of the exocyclic atom N2 were found to be in the plane of the latter and the adjacent six-membered aromatic ring, implying an *sp* ^2^ hybridization of N2. It is of inter­est to note that in the ligand database (Ligand Expo; Feng *et al.*, 2004 ▸) of the Protein Database (Burley *et al.*, 2023 ▸) an incorrect Lewis structure of 2′-de­oxy­guanosine (identifier GNG) is present (Fig. S1 in the supporting information).

## 3. Supra­molecular features

**Figure 2:**
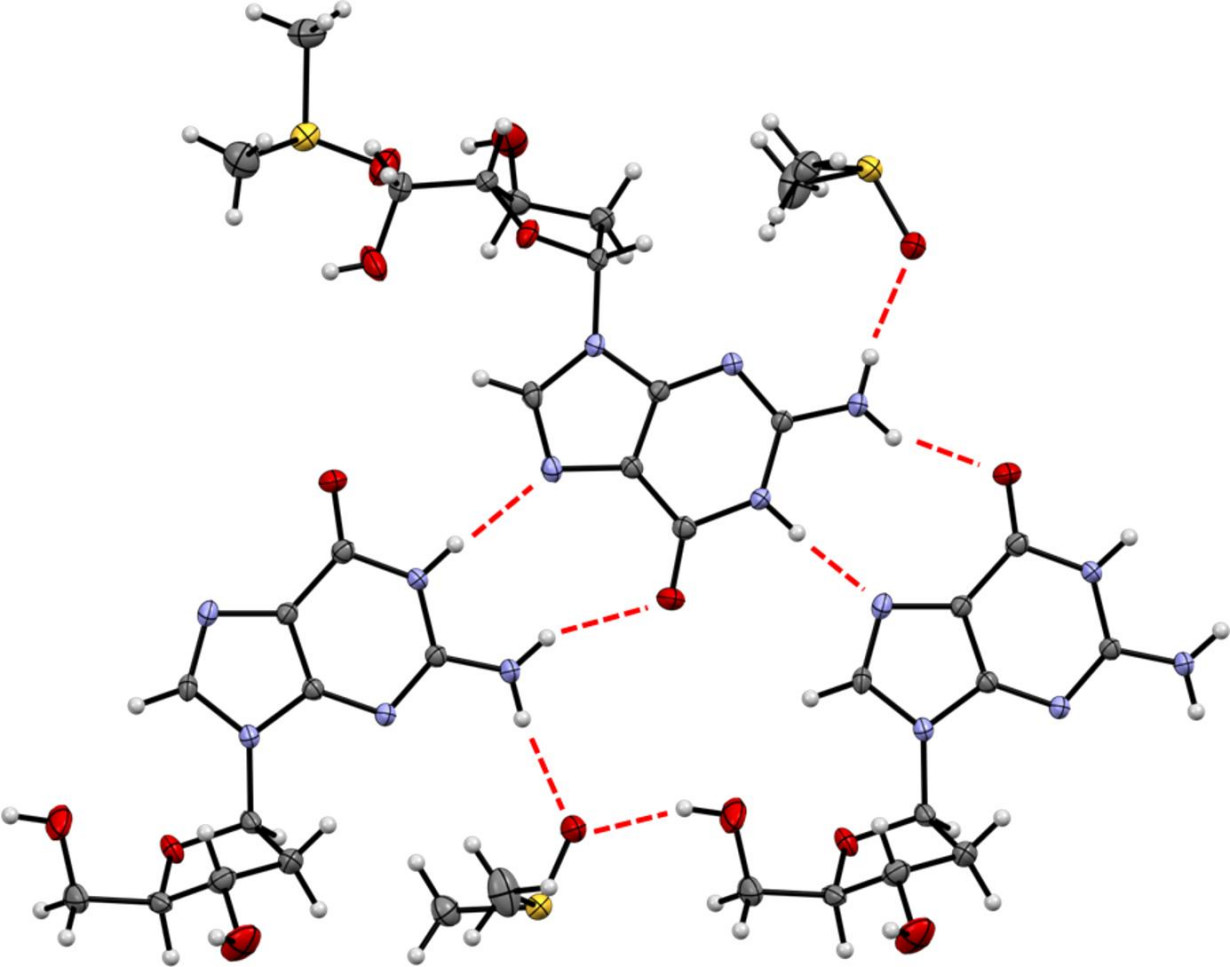
Hydrogen-bonding pattern of (**I**). Displacement ellipsoids are drawn at the 50% probability level.

**Figure 3:**
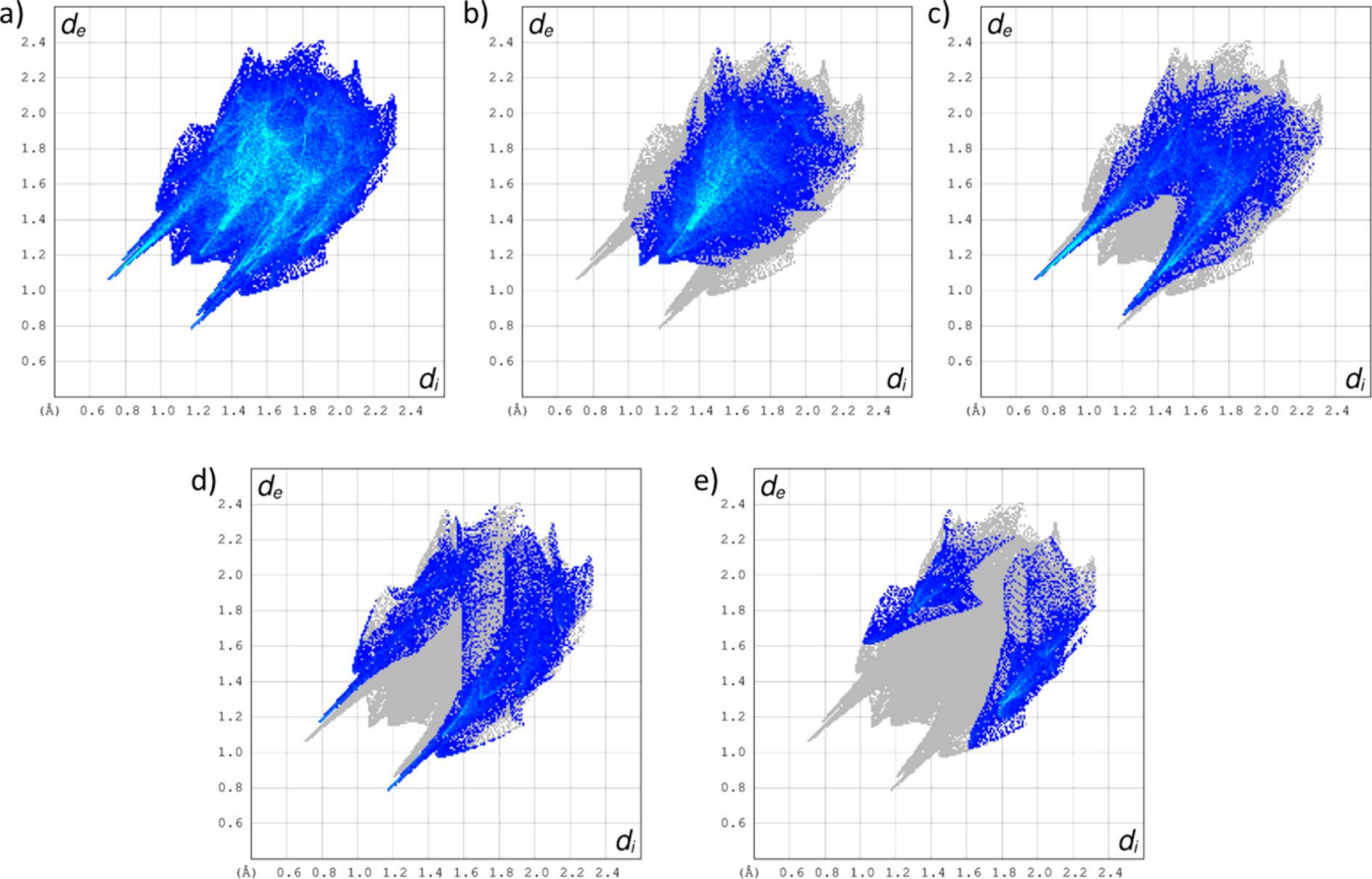
Fingerprint plots of (*a*) the entire Hirshfeld surface of 2′-de­oxy­guanosine within 2′-de­oxy­guanosine·2(DMSO), (*b*) H⋯H contacts, (*c*) O⋯H/H⋯O contacts, (*d*) N⋯H/H⋯N contacts and (*e*) C⋯H/H⋯C contacts.

The hydrogen bonds are listed in Table 2 ▸. The hydrogen bonding among the guanine nucleobases is a reverse Hoogsteen pairing (Johnson *et al.*, 1992 ▸), generating an 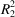 (9) graph set (Bernstein *et al.*, 1995 ▸) (Fig. 2 ▸). A very similar hydrogen-bonding motif was found for guanine monohydrate (Thewalt *et al.*, 1971 ▸) and guanosine dihydrate (Thewalt *et al.*, 1970 ▸). Atom O21 of one DMSO mol­ecule is hydrogen bonded to the secondary alcohol group of 2′-de­oxy­guanosine, while atom O22 of the other DMSO mol­ecule is hydrogen bonded both to the exocyclic amino group of one 2′-de­oxy­guanosine mol­ecule and to the primary –OH group of another 2′-de­oxy­guanosine mol­ecule. Analysis of the fingerprint plots of the Hirshfeld surface around the 2′-de­oxy­guanosine mol­ecule calculated by *CrystalExplorer* (Spackman *et al.*, 2021 ▸) (Fig. 3 ▸) indicates that H⋯H contacts account for 38.3% of the surface contacts, O⋯H/H⋯O contacts for 28.4%, N⋯H/H⋯N for 16.5% and C⋯H/H⋯C for 9.9%.

## 4. Database survey

The search of the Cambridge Structural Database (CSD, Version 5.44, April 2023; Groom *et al.*, 2016 ▸) was made with *ConQuest* (Version 2023.1.0; Bruno *et al.*, 2002 ▸). The first structure containing 2′-de­oxy­guanosine (CSD refcode DGUBCY, a cocrystal with 5-bromo-2′-de­oxy­cytidine) was published by Haschemeyer *et al.* (1965 ▸). However, in the corresponding CSD entry, the Lewis diagram of the 2′-de­oxy­guanosine is wrong (Fig. S2), showing a 2-amino­pyrimidin-4-ol moiety, which should be redrawn as a 2-amino­pyrimidin-4(3*H*)-one. The cocrystal structures of (actinomycin D)·2(2′-de­oxy­guanosine)·12H_2_O (ACTDGU) and (7-bromo­ac­tino­mycin)·2(2′-de­oxy­guanosine)·11H_2_O (BRAXGU) at room temperature were also reported (Sobell *et al.*, 1971 ▸; Jain & Sobell, 1972 ▸). In addition, the X-ray structures of four metal com­plexes containing 2′-de­oxy­guanosine are known (WEWKEO, UKISEM, WUNXIM and EWOBIN; Shionoya *et al.*, 1994 ▸; Ito *et al.*, 2002 ▸; Aoki & Salam, 2002 ▸; Baruah *et al.*, 2004 ▸). Only one of them was recorded at low temperature (Baruah *et al.*, 2004 ▸). Still, even for the latter structure, the average C—C bond distance was determined with a rather low precision of 0.009 Å. The present structure, (**I**), is the only purine nucleoside solvate in the CSD (Groom *et al.*, 2016 ▸) with two DMSO mol­ecules per host mol­ecule.

## 5. Synthesis and crystallization

Single crystals of (**I**) were obtained upon slow evaporation of 2′-de­oxy­guanosine (product number D0052, TCI) from DMSO.

## 6. Refinement

Crystal data, data collection and structure refinement details are summarized in Table 3 ▸. The structure was solved by direct methods with the program *SIR97* (Altomare *et al.*, 1999 ▸). All C-bonded H atoms were placed in ideal positions, with C—H bond lengths of 0.95 Å for aromatic, 1.00 Å for methine, 0.99 Å for methyl­ene and 0.98 Å for methyl C atoms, and refined as riding atoms, except those of methyl group C23H_3_ of one DMSO mol­ecule, which makes a relatively close contact to O6. The latter H atoms and those attached to non-C atoms were freely refined. The *U*_iso_(H) values were set at 1.2 times (for CH, NH, NH_2_ and CH_2_ units) or 1.5 times (for methyl and OH groups) the *U*_eq_ value of the parent atom. The Flack parameter *x* was −0.00 (6) by classical fit to all intensities and 0.022 (14) by Parsons’ method (Parsons *et al.*, 2013 ▸), from 2107 selected quotients.

## 7. Computational details

The sugar conformations (Table 1 ▸) were analysed with *PLATON* (Spek, 2020 ▸), using the published description of such conformations by Saenger (1984 ▸). For older structures, where the CSD does not contain H atoms, these were added using *OLEX2* (Dolomanov *et al.*, 2009 ▸) with default parameters.

## Supplementary Material

Crystal structure: contains datablock(s) I, global. DOI: 10.1107/S2056989023007405/zv2028sup1.cif

Structure factors: contains datablock(s) I. DOI: 10.1107/S2056989023007405/zv2028Isup2.hkl

Figures S1 and S2. DOI: 10.1107/S2056989023007405/zv2028sup3.pdf

CCDC reference: 2290127

Additional supporting information:  crystallographic information; 3D view; checkCIF report

## Figures and Tables

**Table 1:** Sugar conformations in 2′-de­oxy­guanosine, its supra­molecular com­plexes and guanosine

Compound	Space group	Sugar conformation	χ (O4′—C1′—N1—C6) (°)	Reference
2′-De­oxy­guanosine·2(DMSO)	*P*2_1_2_1_2_1_	Envelope, C3′-*endo*	−165.6 (1)	This work
(Actinomycin D)·2(2′-de­oxy­guanosine)·12H_2_O	*P*2_1_2_1_2_1_	Envelope, C3′-*endo*; Twisted, C1′-*exo*/C2′-*endo*	−86.5; −90.6	Jain & Sobell (1972 ▸)
(7-Bromo­actinomycin D)·2(2′-de­oxy­guanosine)·11H_2_O	*P*2_1_2_1_2_1_	Twisted, C2′-*exo*/C3′-*endo*; Twisted, C1′-*exo*/C2′-*endo*	−86.5; −88.9	Jain & Sobell (1972 ▸)
(2′-De­oxy­guanosine)·(5-bromo-2′-de­oxy­cytidine)	*P*2_1_2_1_2	Envelope, C2′-*endo*	56.7	Haschemeyer *et al.* (1965 ▸)
(Guanosine)_2_·4H_2_O	*P*2_1_	Envelope, C2′-*endo*; Twisted, C1′-*exo*/C2′-*endo*	−58.1; −137.2	Thewalt *et al.* (1970 ▸)

**Table 2:** Hydrogen-bond geometry (Å, °)

*D*—H⋯*A*	*D*—H	H⋯*A*	*D*⋯*A*	*D*—H⋯*A*
C23—H23*B*⋯O6^i^	0.96 (3)	2.35 (3)	3.239 (3)	153 (2)
N1—H1⋯N7^ii^	0.87 (2)	2.09 (2)	2.962 (2)	173 (2)
N2—H2*A*⋯O22	0.83 (2)	2.09 (2)	2.902 (2)	168 (2)
N2—H2*B*⋯O6^ii^	0.83 (2)	2.24 (2)	3.012 (2)	155 (2)
O13—H13*A*⋯O21	0.87 (3)	1.88 (4)	2.738 (3)	169 (3)
O15—H15⋯O22^iii^	0.89 (3)	1.91 (3)	2.752 (2)	159 (3)

Symmetry codes: (i) 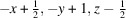 ; (ii) 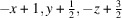 ; (iii) 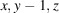 .

**Table 3:** Experimental details

Crystal data	
Chemical formula	C_10_H_13_N_5_O_4_·2C_2_H_6_OS
*M*_r_	423.51
Crystal system, space group	Orthorhombic, *P*2_1_2_1_2_1_
Temperature (K)	183
*a*, *b*, *c* (Å)	9.7590 (1), 11.7951 (2), 16.7553 (2)
*V* (Å^3^)	1928.68 (4)
*Z*	4
Radiation type	Mo *K*α
μ (mm^−1^)	0.32
Crystal size (mm)	0.44 × 0.24 × 0.17

Data collection	
Diffractometer	Oxford Diffraction Xcalibur Ruby
Absorption correction	Multi-scan (*CrysAlis RED*; Oxford Diffraction, 2008 ▸)
*T*_min_, *T*_max_	0.909, 1.000
No. of measured, independent and observed [*I* > 2σ(*I*)] reflections	19896, 5874, 5295
*R*_int_	0.024
(sin θ/λ)_max_ (Å^−1^)	0.714

Refinement	
*R*[*F* ^2^ > 2σ(*F* ^2^)], *wR*(*F* ^2^), *S*	0.030, 0.073, 1.01
No. of reflections	5874
No. of parameters	271
H-atom treatment	H atoms treated by a mixture of independent and constrained refinement
Δρ_max_, Δρ_min_ (e Å^−3^)	0.30, −0.27
Absolute structure	Flack *x* determined using 2107 quotients [(*I* ^+^) − (*I* ^−^)]/[(*I* ^+^) + (*I* ^−^)] (Parsons *et al.*, 2013 ▸)
Absolute structure parameter	0.022 (14)

Computer programs: *CrysAlis CCD* (Oxford Diffraction, 2008 ▸), *CrysAlis RED* (Oxford Diffraction, 2008 ▸), *SIR97* (Altomare *et al.*, 1999 ▸), *SHELXL2018* (Sheldrick, 2015 ▸) and *OLEX2* (Dolomanov *et al.*, 2009 ▸).

## supplementary crystallographic information

### Crystal data

**Table:**

C_10_H_13_N_5_O_4_·2C_2_H_6_OS	*D*_x_ = 1.459 Mg m^−^^3^
*M**_r_* = 423.51	Mo *K*α radiation, λ = 0.71073 Å
Orthorhombic, *P*2_1_2_1_2_1_	Cell parameters from 11856 reflections
*a* = 9.7590 (1) Å	θ = 2.4–32.7°
*b* = 11.7951 (2) Å	µ = 0.32 mm^−^^1^
*c* = 16.7553 (2) Å	*T* = 183 K
*V* = 1928.68 (4) Å^3^	Needle, colourless
*Z* = 4	0.44 × 0.24 × 0.17 mm
*F*(000) = 896	

### Data collection

**Table:**

Oxford Diffraction Xcalibur Ruby diffractometer	5874 independent reflections
Radiation source: Enhance (Mo) X-ray Source	5295 reflections with *I* > 2σ(*I*)
Graphite monochromator	*R*_int_ = 0.024
Detector resolution: 10.4498 pixels mm^-1^	θ_max_ = 30.5°, θ_min_ = 2.4°
ω scans	*h* = −13→13
Absorption correction: multi-scan (CrysAlis RED; Oxford Diffraction, 2008)	*k* = −14→16
*T*_min_ = 0.909, *T*_max_ = 1.000	*l* = −22→23
19896 measured reflections	

### Refinement

**Table:**

Refinement on *F*^2^	Hydrogen site location: mixed
Least-squares matrix: full	H atoms treated by a mixture of independent and constrained refinement
*R*[*F*^2^ > 2σ(*F*^2^)] = 0.030	*w* = 1/[σ^2^(*F*_o_^2^) + (0.045*P*)^2^] where *P* = (*F*_o_^2^ + 2*F*_c_^2^)/3
*wR*(*F*^2^) = 0.073	(Δ/σ)_max_ < 0.001
*S* = 1.01	Δρ_max_ = 0.30 e Å^−^^3^
5874 reflections	Δρ_min_ = −0.27 e Å^−^^3^
271 parameters	Absolute structure: Flack *x* determined using 2107 quotients [(I+)-(I-)]/[(I+)+(I-)] (Parsons *et al.*, 2013)
0 restraints	Absolute structure parameter: 0.022 (14)
Primary atom site location: structure-invariant direct methods	

### Special details

**Table:**

Geometry. All esds (except the esd in the dihedral angle between two l.s. planes) are estimated using the full covariance matrix. The cell esds are taken into account individually in the estimation of esds in distances, angles and torsion angles; correlations between esds in cell parameters are only used when they are defined by crystal symmetry. An approximate (isotropic) treatment of cell esds is used for estimating esds involving l.s. planes.

### Fractional atomic coordinates and isotropic or equivalent isotropic displacement parameters (Å^2^)

**Table:**

	*x*	*y*	*z*	*U*_iso_*/*U*_eq_	
C2	0.35188 (19)	0.60776 (14)	0.66640 (9)	0.0159 (3)	
C4	0.31935 (18)	0.42499 (14)	0.64001 (9)	0.0157 (3)	
C5	0.40248 (18)	0.38572 (15)	0.70072 (10)	0.0163 (3)	
C6	0.47568 (18)	0.46616 (14)	0.74730 (10)	0.0183 (3)	
C8	0.32641 (18)	0.23836 (15)	0.64098 (10)	0.0184 (3)	
H8	0.308547	0.161923	0.626466	0.022*	
C11	0.17747 (19)	0.33364 (15)	0.53323 (10)	0.0192 (3)	
H11	0.101178	0.388127	0.543999	0.023*	
C12	0.2513 (2)	0.36613 (16)	0.45639 (10)	0.0242 (4)	
H12A	0.190773	0.410232	0.420577	0.029*	
H12B	0.334890	0.410942	0.467733	0.029*	
C13	0.2870 (2)	0.25191 (18)	0.42025 (10)	0.0243 (4)	
H13	0.370902	0.219466	0.445824	0.029*	
C14	0.16057 (19)	0.18386 (15)	0.44271 (11)	0.0212 (4)	
H14	0.084806	0.204527	0.405249	0.025*	
C15	0.1745 (2)	0.05766 (16)	0.44360 (13)	0.0296 (4)	
H15A	0.088134	0.023128	0.462851	0.035*	
H15B	0.191768	0.029894	0.388760	0.035*	
C23	0.0397 (2)	0.63289 (18)	0.47584 (13)	0.0280 (4)	
H23A	0.093 (3)	0.593 (2)	0.5123 (15)	0.042*	
H23B	0.010 (3)	0.585 (2)	0.4328 (16)	0.042*	
H23C	−0.038 (3)	0.668 (2)	0.4972 (15)	0.042*	
C24	0.2803 (3)	0.6683 (2)	0.40425 (16)	0.0438 (6)	
H24A	0.248599	0.617530	0.361775	0.066*	
H24B	0.319500	0.623323	0.447802	0.066*	
H24C	0.350235	0.719659	0.382971	0.066*	
N1	0.44138 (16)	0.57763 (13)	0.72580 (8)	0.0173 (3)	
H1	0.483 (2)	0.6326 (19)	0.7512 (13)	0.021*	
N2	0.32939 (19)	0.71824 (13)	0.65567 (9)	0.0218 (3)	
H2A	0.281 (3)	0.737 (2)	0.6175 (13)	0.026*	
H2B	0.367 (3)	0.771 (2)	0.6808 (13)	0.026*	
N3	0.28910 (16)	0.53300 (12)	0.61976 (8)	0.0169 (3)	
N7	0.40557 (16)	0.26752 (12)	0.70054 (8)	0.0187 (3)	
N9	0.27173 (15)	0.33028 (12)	0.60156 (8)	0.0168 (3)	
O6	0.56048 (15)	0.44860 (12)	0.80073 (7)	0.0270 (3)	
O13	0.3041 (2)	0.26086 (16)	0.33678 (8)	0.0432 (4)	
H13A	0.363 (4)	0.215 (3)	0.3158 (18)	0.065*	
O14	0.12431 (14)	0.22319 (10)	0.52196 (7)	0.0219 (3)	
O15	0.28382 (17)	0.02499 (13)	0.49400 (10)	0.0385 (4)	
H15	0.275 (3)	−0.048 (3)	0.5060 (16)	0.058*	
O21	0.50822 (19)	0.11673 (15)	0.28913 (10)	0.0440 (4)	
O22	0.19120 (17)	0.80671 (12)	0.51554 (8)	0.0318 (3)	
S22	0.13986 (5)	0.74863 (4)	0.44083 (3)	0.02532 (11)	
S21	0.53507 (5)	0.01236 (4)	0.24068 (3)	0.02556 (11)	
C22	0.4129 (2)	0.0115 (2)	0.16179 (12)	0.0362 (5)	
H22A	0.431974	0.074434	0.125214	0.054*	
H22B	0.320604	0.020153	0.183990	0.054*	
H22C	0.418952	−0.060392	0.132742	0.054*	
C21	0.4665 (3)	−0.1037 (2)	0.29503 (13)	0.0396 (5)	
H21A	0.371508	−0.087110	0.309943	0.059*	
H21B	0.521108	−0.116028	0.343349	0.059*	
H21C	0.469085	−0.172052	0.261807	0.059*	

### Atomic displacement parameters (Å^2^)

**Table:**

	*U*^11^	*U*^22^	*U*^33^	*U*^12^	*U*^13^	*U*^23^
C2	0.0194 (8)	0.0140 (8)	0.0144 (7)	0.0000 (6)	0.0000 (6)	−0.0005 (6)
C4	0.0175 (8)	0.0146 (8)	0.0150 (7)	−0.0013 (6)	0.0000 (6)	−0.0009 (6)
C5	0.0184 (8)	0.0146 (8)	0.0158 (7)	0.0009 (6)	−0.0004 (6)	0.0015 (6)
C6	0.0216 (8)	0.0172 (8)	0.0160 (7)	0.0014 (7)	0.0003 (7)	0.0009 (6)
C8	0.0208 (8)	0.0115 (8)	0.0231 (8)	−0.0016 (7)	0.0007 (6)	0.0026 (6)
C11	0.0211 (9)	0.0129 (8)	0.0235 (8)	0.0001 (6)	−0.0063 (7)	−0.0033 (6)
C12	0.0327 (10)	0.0191 (9)	0.0207 (8)	−0.0027 (8)	−0.0048 (7)	0.0022 (7)
C13	0.0301 (9)	0.0238 (9)	0.0189 (7)	0.0038 (8)	−0.0012 (7)	0.0018 (7)
C14	0.0247 (9)	0.0165 (9)	0.0224 (8)	0.0042 (7)	−0.0097 (7)	−0.0051 (7)
C15	0.0368 (11)	0.0169 (9)	0.0350 (10)	0.0027 (8)	−0.0101 (9)	−0.0059 (8)
C23	0.0255 (10)	0.0275 (11)	0.0310 (10)	−0.0040 (9)	0.0012 (9)	−0.0017 (8)
C24	0.0400 (13)	0.0350 (13)	0.0564 (14)	−0.0071 (11)	0.0217 (11)	−0.0083 (11)
N1	0.0228 (8)	0.0124 (7)	0.0166 (6)	−0.0014 (6)	−0.0055 (6)	−0.0015 (5)
N2	0.0317 (9)	0.0118 (7)	0.0218 (7)	−0.0004 (6)	−0.0091 (7)	−0.0011 (6)
N3	0.0208 (7)	0.0127 (7)	0.0171 (6)	0.0010 (6)	−0.0035 (5)	−0.0001 (5)
N7	0.0223 (7)	0.0125 (7)	0.0212 (7)	0.0005 (6)	0.0001 (6)	0.0032 (5)
N9	0.0191 (7)	0.0120 (7)	0.0195 (6)	−0.0018 (6)	−0.0031 (5)	−0.0003 (5)
O6	0.0339 (8)	0.0236 (7)	0.0234 (6)	0.0003 (6)	−0.0129 (6)	0.0019 (5)
O13	0.0663 (12)	0.0431 (10)	0.0201 (7)	0.0143 (9)	0.0066 (7)	0.0027 (7)
O14	0.0225 (6)	0.0156 (6)	0.0275 (6)	−0.0048 (5)	−0.0010 (5)	−0.0059 (5)
O15	0.0365 (8)	0.0198 (8)	0.0592 (10)	0.0036 (7)	−0.0145 (7)	0.0086 (7)
O21	0.0483 (11)	0.0361 (9)	0.0477 (10)	0.0013 (8)	−0.0043 (8)	−0.0179 (7)
O22	0.0463 (9)	0.0188 (7)	0.0302 (7)	−0.0004 (7)	−0.0144 (7)	−0.0014 (5)
S22	0.0316 (2)	0.0205 (2)	0.0239 (2)	−0.0008 (2)	−0.00666 (18)	0.00218 (18)
S21	0.0255 (2)	0.0264 (3)	0.0248 (2)	−0.00201 (19)	−0.00031 (19)	−0.00260 (17)
C22	0.0353 (11)	0.0424 (13)	0.0310 (10)	−0.0071 (10)	−0.0079 (9)	0.0039 (9)
C21	0.0464 (14)	0.0379 (13)	0.0345 (11)	−0.0042 (11)	0.0051 (11)	0.0076 (9)

### Geometric parameters (Å, º)

**Table:**

C2—N1	1.371 (2)	C15—H15A	0.9900
C2—N2	1.334 (2)	C15—H15B	0.9900
C2—N3	1.328 (2)	C15—O15	1.414 (2)
C4—C5	1.381 (2)	C23—H23A	0.93 (3)
C4—N3	1.351 (2)	C23—H23B	0.96 (3)
C4—N9	1.371 (2)	C23—H23C	0.94 (3)
C5—C6	1.421 (2)	C23—S22	1.778 (2)
C5—N7	1.395 (2)	C24—H24A	0.9800
C6—N1	1.404 (2)	C24—H24B	0.9800
C6—O6	1.237 (2)	C24—H24C	0.9800
C8—H8	0.9500	C24—S22	1.775 (2)
C8—N7	1.308 (2)	N1—H1	0.87 (2)
C8—N9	1.377 (2)	N2—H2A	0.83 (2)
C11—H11	1.0000	N2—H2B	0.83 (2)
C11—C12	1.525 (2)	O13—H13A	0.87 (3)
C11—N9	1.469 (2)	O15—H15	0.89 (3)
C11—O14	1.415 (2)	O21—S21	1.4978 (17)
C12—H12A	0.9900	O22—S22	1.5124 (14)
C12—H12B	0.9900	S21—C22	1.780 (2)
C12—C13	1.518 (3)	S21—C21	1.775 (2)
C13—H13	1.0000	C22—H22A	0.9800
C13—C14	1.520 (3)	C22—H22B	0.9800
C13—O13	1.412 (2)	C22—H22C	0.9800
C14—H14	1.0000	C21—H21A	0.9800
C14—C15	1.495 (3)	C21—H21B	0.9800
C14—O14	1.450 (2)	C21—H21C	0.9800
			
N2—C2—N1	117.13 (15)	H23A—C23—H23B	111 (2)
N3—C2—N1	123.30 (15)	H23A—C23—H23C	115 (2)
N3—C2—N2	119.57 (16)	H23B—C23—H23C	108 (2)
N3—C4—C5	129.01 (15)	S22—C23—H23A	107.2 (17)
N3—C4—N9	125.20 (15)	S22—C23—H23B	111.7 (15)
N9—C4—C5	105.78 (15)	S22—C23—H23C	103.7 (16)
C4—C5—C6	118.42 (16)	H24A—C24—H24B	109.5
C4—C5—N7	110.26 (15)	H24A—C24—H24C	109.5
N7—C5—C6	131.13 (16)	H24B—C24—H24C	109.5
N1—C6—C5	111.38 (14)	S22—C24—H24A	109.5
O6—C6—C5	128.47 (16)	S22—C24—H24B	109.5
O6—C6—N1	120.15 (15)	S22—C24—H24C	109.5
N7—C8—H8	123.6	C2—N1—C6	125.52 (14)
N7—C8—N9	112.82 (15)	C2—N1—H1	117.2 (14)
N9—C8—H8	123.6	C6—N1—H1	117.3 (15)
C12—C11—H11	110.1	C2—N2—H2A	117.4 (17)
N9—C11—H11	110.1	C2—N2—H2B	125.9 (17)
N9—C11—C12	111.64 (15)	H2A—N2—H2B	116 (2)
O14—C11—H11	110.1	C2—N3—C4	112.18 (14)
O14—C11—C12	106.99 (14)	C8—N7—C5	104.58 (15)
O14—C11—N9	107.98 (14)	C4—N9—C8	106.55 (13)
C11—C12—H12A	111.2	C4—N9—C11	123.81 (14)
C11—C12—H12B	111.2	C8—N9—C11	129.61 (14)
H12A—C12—H12B	109.1	C13—O13—H13A	116 (2)
C13—C12—C11	102.84 (15)	C11—O14—C14	109.10 (14)
C13—C12—H12A	111.2	C15—O15—H15	109 (2)
C13—C12—H12B	111.2	C24—S22—C23	97.37 (11)
C12—C13—H13	110.9	O22—S22—C23	104.88 (9)
C12—C13—C14	100.59 (15)	O22—S22—C24	105.77 (12)
C14—C13—H13	110.9	O21—S21—C22	106.85 (11)
O13—C13—C12	110.84 (16)	O21—S21—C21	106.85 (11)
O13—C13—H13	110.9	C21—S21—C22	97.15 (12)
O13—C13—C14	112.36 (17)	S21—C22—H22A	109.5
C13—C14—H14	108.4	S21—C22—H22B	109.5
C15—C14—C13	117.04 (17)	S21—C22—H22C	109.5
C15—C14—H14	108.4	H22A—C22—H22B	109.5
O14—C14—C13	104.83 (14)	H22A—C22—H22C	109.5
O14—C14—H14	108.4	H22B—C22—H22C	109.5
O14—C14—C15	109.36 (16)	S21—C21—H21A	109.5
C14—C15—H15A	109.6	S21—C21—H21B	109.5
C14—C15—H15B	109.6	S21—C21—H21C	109.5
H15A—C15—H15B	108.1	H21A—C21—H21B	109.5
O15—C15—C14	110.24 (16)	H21A—C21—H21C	109.5
O15—C15—H15A	109.6	H21B—C21—H21C	109.5
O15—C15—H15B	109.6		
			
C4—C5—C6—N1	4.3 (2)	N3—C4—C5—C6	−4.4 (3)
C4—C5—C6—O6	−175.47 (17)	N3—C4—C5—N7	−179.94 (17)
C4—C5—N7—C8	0.2 (2)	N3—C4—N9—C8	−179.89 (17)
C5—C4—N3—C2	0.6 (3)	N3—C4—N9—C11	−1.5 (3)
C5—C4—N9—C8	0.69 (18)	N7—C5—C6—N1	178.83 (18)
C5—C4—N9—C11	179.13 (15)	N7—C5—C6—O6	−1.0 (3)
C5—C6—N1—C2	−1.6 (2)	N7—C8—N9—C4	−0.6 (2)
C6—C5—N7—C8	−174.68 (18)	N7—C8—N9—C11	−178.96 (16)
C11—C12—C13—C14	36.85 (17)	N9—C4—C5—C6	175.04 (15)
C11—C12—C13—O13	155.88 (17)	N9—C4—C5—N7	−0.55 (19)
C12—C11—N9—C4	77.0 (2)	N9—C4—N3—C2	−178.67 (16)
C12—C11—N9—C8	−104.9 (2)	N9—C8—N7—C5	0.3 (2)
C12—C11—O14—C14	0.18 (18)	N9—C11—C12—C13	93.88 (17)
C12—C13—C14—C15	−159.00 (16)	N9—C11—O14—C14	−120.12 (15)
C12—C13—C14—O14	−37.66 (16)	O6—C6—N1—C2	178.24 (16)
C13—C14—C15—O15	55.1 (2)	O13—C13—C14—C15	83.1 (2)
C13—C14—O14—C11	23.95 (18)	O13—C13—C14—O14	−155.58 (16)
C15—C14—O14—C11	150.21 (15)	O14—C11—C12—C13	−24.05 (18)
N1—C2—N3—C4	2.6 (2)	O14—C11—N9—C4	−165.65 (15)
N2—C2—N1—C6	178.46 (17)	O14—C11—N9—C8	12.4 (2)
N2—C2—N3—C4	−178.00 (16)	O14—C14—C15—O15	−63.8 (2)
N3—C2—N1—C6	−2.1 (3)		

### Hydrogen-bond geometry (Å, º)

**Table:**

*D*—H···*A*	*D*—H	H···*A*	*D*···*A*	*D*—H···*A*
C23—H23*B*···O6^i^	0.96 (3)	2.35 (3)	3.239 (3)	153 (2)
N1—H1···N7^ii^	0.87 (2)	2.09 (2)	2.962 (2)	173 (2)
N2—H2*A*···O22	0.83 (2)	2.09 (2)	2.902 (2)	168 (2)
N2—H2*B*···O6^ii^	0.83 (2)	2.24 (2)	3.012 (2)	155 (2)
O13—H13*A*···O21	0.87 (3)	1.88 (4)	2.738 (3)	169 (3)
O15—H15···O22^iii^	0.89 (3)	1.91 (3)	2.752 (2)	159 (3)

Symmetry codes: (i) −*x*+1/2, −*y*+1, *z*−1/2; (ii) −*x*+1, *y*+1/2, −*z*+3/2; (iii) *x*, *y*−1, *z*.
