# Supplementary material for: Crystal structure of the nucleoside 2′-de­oxy­guanosine dimethyl sulfoxide disolvate
Source: Acta Crystallogr E Crystallogr Commun. 2023 Aug 30;79(Pt 9):852–5. doi: 10.1107/S2056989023007405 (PMC10483554; doi:10.1107/S2056989023007405)
Supplement: Supplementary file 3 [file e-79-00852-sup3.pdf]

Supporting information for

Crystal structure of the nucleoside 2’-deoxyguanosine

Bernhard Spingler*

Department of Chemistry, University of Zurich, Winterthurerstr 190, Zürich, 8057, Switzerland

Correspondence email: spingler@chem.uzh.ch

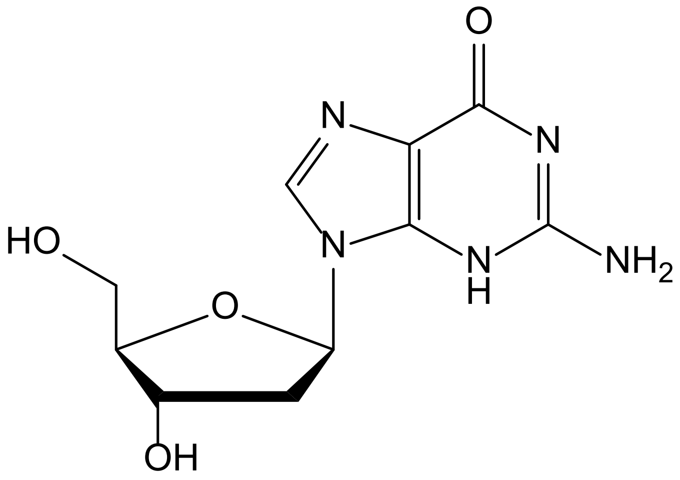

Figure S1 Incorrect Lewis structure of 2’-deoxyguanosine, which is used as a reference structure (identifier GNG) in the ligand database (Ligand Expo) of the Protein Database.

IMPORTANT: this document contains embedded data - to preserve data integrity, please ensure where possible that the IUCr Word tools (available from http://journals.iucr.org/services/docxtemplate/) are installed when editing this document.

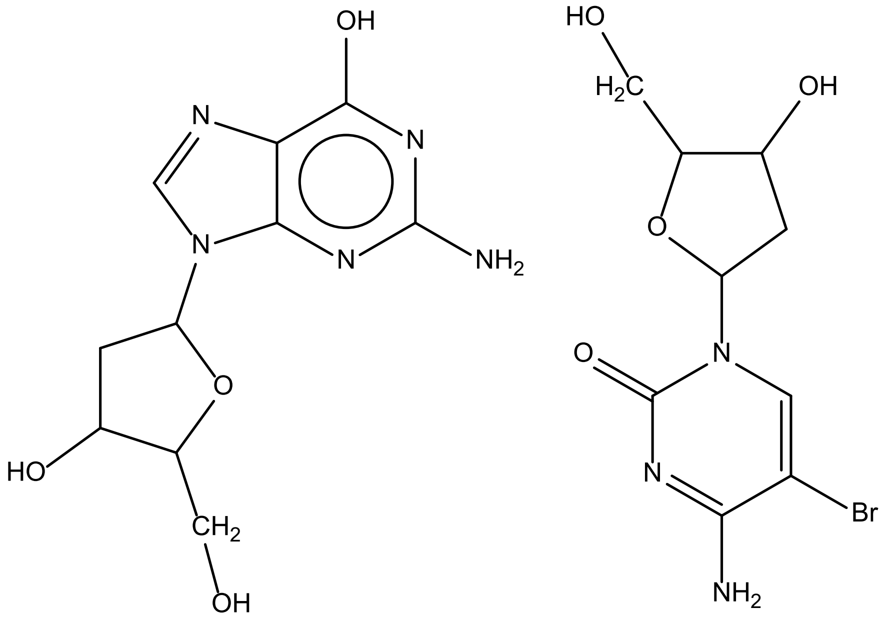

Figure S2 Incorrectly drawn Lewis structure in the Cambridge Structure Database (CSD version 5.44, April 2023) of entry DGUBCY (2'-deoxyguanosine together with 5-bromo-2'-deoxycytidine).
